# Wearable Nanocomposite Sensor System for Motion Phenotyping Chronic Low Back Pain: A BACPAC Technology Research Site

**DOI:** 10.1093/pm/pnad017

**Published:** 2023-02-17

**Authors:** Spencer A Baker, Darci A Billmire, R Adam Bilodeau, Darian Emmett, Andrew K Gibbons, Ulrike H Mitchell, Anton E Bowden, David T Fullwood

**Affiliations:** Department of Mechanical Engineering, Brigham Young University, Provo, UT, United States; Department of Mechanical Engineering, Brigham Young University, Provo, UT, United States; Department of Mechanical Engineering, Brigham Young University, Provo, UT, United States; Department of Mechanical Engineering, Brigham Young University, Provo, UT, United States; Department of Mechanical Engineering, Brigham Young University, Provo, UT, United States; Department of Exercise Sciences, Brigham Young University, Provo, UT, United States; Department of Mechanical Engineering, Brigham Young University, Provo, UT, United States; Department of Mechanical Engineering, Brigham Young University, Provo, UT, United States

**Keywords:** chronic low back pain, machine learning, phenotypes, clusters, spinal motion, optimization

## Abstract

Chronic low back pain (cLBP) is a prevalent and multifactorial ailment. No single treatment has been shown to dramatically improve outcomes for all cLBP patients, and current techniques of linking a patient with their most effective treatment lack validation. It has long been recognized that spinal pathology alters motion. Therefore, one potential method to identify optimal treatments is to evaluate patient movement patterns (ie, motion-based phenotypes). Biomechanists, physical therapists, and surgeons each utilize a variety of tools and techniques to qualitatively assess movement as a critical element in their treatment paradigms. However, objectively characterizing and communicating this information is challenging due to the lack of economical, objective, and accurate clinical tools. In response to that need, we have developed a wearable array of nanocomposite stretch sensors that accurately capture the lumbar spinal kinematics, the SPINE Sense System. Data collected from this device are used to identify movement-based phenotypes and analyze correlations between spinal kinematics and patient-reported outcomes.

The purpose of this paper is twofold: first, to describe the design and validity of the SPINE Sense System; and second, to describe the protocol and data analysis toward the application of this equipment to enhance understanding of the relationship between spinal movement patterns and patient metrics, which will facilitate the identification of optimal treatment paradigms for cLBP.

## Introduction

Low back pain (LBP) is a prevalent ailment. In the United States alone, it is estimated that 80% of the population will experience a back problem at some point in their lives,^1^ and that at any given time 31 million Americans are suffering from LBP.^2^ Not only is LBP common, but it also has the potential to be very severe. Back pain is the single leading cause of disability.^3^ Treating back pain imposes a significant economic cost as well. In the United States alone, over $100 billion are annually lost from treatment costs and productivity losses associated with LBP.^4^ Unfortunately, the increased costs of treating lower back pain have not mitigated the issue.^5^

The ineffectiveness of LBP treatments is due in part to the limitations in current diagnostic techniques. The most common diagnostic methods employ static imaging (eg, computed tomography [CT] or magnetic resonance imaging [MRI] scans) to detect anatomical anomalies. However, it is difficult to pinpoint the etiology of chronic and other types of non-specific low back pain (cLBP) using these methods.^6^ Virtually all static images are acquired with the patient lying down or standing, failing to capture the dynamic aspect of lower back pain which could be critical to the diagnosis. Furthermore, many patients suffering from cLBP do not exhibit clear anatomical anomalies in their spine.^7^ Rather, the pain is the result of myriad psychological, social, and biological factors;^8^ additionally, many subjects who exhibit anatomical anomalies do not experience corresponding symptoms, indicating that anatomical anomalies are often unrelated to the underlying cause of the subject’s pain.^9^ In summary, it is very difficult to prescribe treatments that will resolve the underlying cause of cLBP because those underlying causes might have been resolved, or in case that they are still present, cannot be captured with static images or existing biomarkers.

Several methods and many studies have attempted to identify linkages between patients and their most effective treatment paradigm. One intriguing approach is to evaluate patient motion patterns (ie, movement phenotypes). It is well known that individuals with acute and chronic back pain move differently than asymptomatic controls, a phenomenon that potentially becomes more pronounced the longer the symptoms last.^10–12^ Patients intrinsically recognize motions and postures that cause pain and alter their position accordingly. Usually, a reduction in spinal range of motion in uniplanar^13^^,^^14^ and multiplanar directions^15^ is involved. Biomechanists, physical therapists, chiropractors, and surgeons each utilize a variety of tools and techniques to assess and interpret qualitative movement changes as a window to understanding potential mechanical and neurological sources of low back pain, and as a critical element in their treatment paradigm. It is therefore reasonable to postulate that motion-based phenotyping of cLBP could be valuable in finding effective treatment options^16^ and in evaluating patients’ rehabilitation progress. Past studies that have investigated phenotyping as a means to identify optimal LBP treatments and have found that while several classifications methods show promise, the margin of improved treatment outcomes currently fall short of clinical significance.^17^ These and related studies often rely on qualitative assessments of LBP symptoms^18^ or highly trained individuals^19^ to classify patients according to their respective phenotypes, making these methods liable to misdiagnosis due to either the subjective nature of the assessment^17^^,^^20^ or clinical inexperience.^21^

Overall, the identification of LBP classes—particularly those that account for spinal-motion phenotypes—is a concept that shows enormous potential, but is stymied by the lack of cost-effective, objective, and accurate tools that are compatible with the clinical setting. A valid and reliable quantitative assessment tool is therefore necessary to objectively measure and monitor spinal motion^22^ in order to identify and analyze said phenotypes. Our BACPAC Technology Research Site team has addressed this challenge through the use of unique, inexpensive, elastomer-based nano-composite piezoresponsive sensors. Specifically, we have integrated these sensors into a SPInal Nanosensor Environment (SPINE Sense System), which provides an objective, quantitative platform for diagnosis, monitoring, and follow-up assessment of movement changes associated with cLBP. The raw data that are collected from the subjects are comprised of electrical signals from an array of these piezoresponsive sensors that are optimally arranged on the skin of the lower back. These sensors measure local skin strains, which, in turn, correlate with the kinematic motion of the lumbar spine.^23^^,^^24^ This method of using externally mounted sensors offers advantages over alternative kinematic-measuring methods, such as percutaneous skeletal trackers or radiographic imaging, which either involve invasive tools or extended exposure to radiation.^23^ It is also a more economical solution than other motion-capture techniques such as optical cameras, which require expensive equipment and elaborate set up. These sensors have already been used in several biomechanical applications, such as estimating knee kinematics,^25^ calculating ground reaction forces,^26^ measuring ligament strain,^27^ estimating range of joint motion,^28^ tracking pulse,^29^ evaluating upper limb posture,^30^ and monitoring fetal movements,^31^ with promising results.

To make this technology applicable to biomechanical phenotyping of the spine, our laboratory has engaged in multiple phases. The overall purpose of this paper is to describe the completed and promising phases of our work.

- In the section “system design and validation,” we will describe our completed work focused on the design and development of our SPINE Sense array, along with our experiments that describe the validity, reliability, and usability of our equipment to capture spinal kinematics objectively.
- The section “subject testing protocol” presents current step of our BACPAC study, describing our current human subject testing protocol.
- The section “data analysis” describes how the data collected from this protocol will be analyzed to leverage spinal kinematics to determine to what extent they are related to patient-reported outcomes (PROs) and how they can be leveraged to identify dominant motion phenotypes. Some preliminary findings from the early stages of data collection are also presented in this section. It is expected that the outcomes from these analyses will lead to enhanced diagnostics, patient monitoring, and eventually to more effective clinical treatment paradigms.^21^

**Figure 1.**
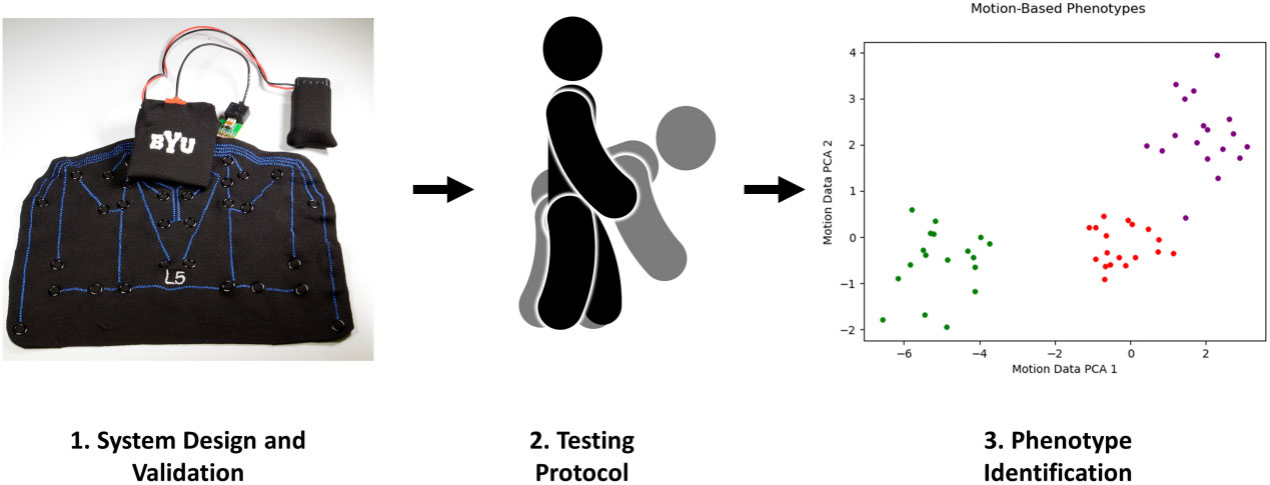
Overview of methodology to identify and analyze movement-based phenotypes among symptomatic and asymptomatic subjects by (1) developing and validation system design (2) collecting data from symptomatic and asymptomatic subjects during several single-planar and multi-planar spinal motions, and (3) analyzing the spinal motion data to identify and interpret different motion-based phenotypes among subjects.

These three phases of research and development are depicted in Figure 1.

## System design and validation

The primary objective of this article is to present the protocol that is being used to collect spinal motion data from control participants and participants with cLBP subjects and the proposed methods to analyze those data to determine the correlation between spinal motion and PROs. We previously reported on the development of the SPINE Sense System which is used to effectively monitor spinal motion. A brief recap of the elements of that design will follow (see also references^24^^,^^32–34^ for additional information).

- Array design: To obtain the clearest reading from the sensors and an accurate estimation of spinal kinematics, the skin-mounted strain gauges were optimally placed on the lines of maximum extension (LoME) of the skin.
- Array implementation: A prototype of the strain gauge array to be used in clinical applications was presented to prospective users. The usability of the system was evaluated, and suggestions were recorded to improve future iterations of the design.
- Electronics design: A key aspect of the system was to provide patients and clinicians with a user-friendly device that can monitor spinal motion. An app was designed to collect and store the data in an efficient manner and provide the users with an intuitive interface.

### Array design

In previous endeavors, skin-mountable nanocomposite sensors (composed of nickel nano strands and nickel coated carbon fibers embedded in a silicone matrix) have been developed by researchers at Brigham Young University.^35^ In this endear, these sensors are being used to detect the skin strain in the area of the lumbar spine to obtain an estimate of the underlying spinal kinematics (related studies have also used skin-mounted sensors to obtain estimates of knee^36^ and spinal motions^37^). In order to extract an accurate estimate of spinal kinematics, the sensors must be strategically placed to detect skin stretch during different motions. Specifically, we desire to estimate the primary kinematics of the spine, which are comprised of flexion-extension along the sagittal plane, lateral bending (left and right) along the coronal plane, and axial rotation (clockwise and counterclockwise) along the axial plane.^38^^,^^39^ In a related study, researchers seeking to optimize the placement of skin-mounted motion capture markers to estimate knee kinematics approached this task by placing sensors along the lines of maximum extension (LoME) during different motions,^40^ which facilitated accurate knee kinematic estimations along multiple degrees of freedom.^36^

In order to determine the LoME, spinal motion and skin elongation data were extracted during a motion-capture experiment.^41^ Data were collected from 28 subjects. This sample size was selected to provide sufficient distinguishing power, accounting for differences between genders and other subject demographics, and is consistent with or exceeds the sample sizes used in related studies.^23^^,^^37^ A summary of the subject demographics of the participants in this study are shown in Table 1.

**Table 1.** Summary of the subject demographics in motion-marker study (the average of each demographic and the corresponding standard deviation of the motion capture participants)

Demographic	
Age (years) [SD]	25.3 [9.2]
Height (cm) [SD]	176.5 [8.4]
Weight (kg) [SD]	73.6 [14.24]
BMI (kg/m^3^) [SD]	23.5 [3.3]
M/F	15/13

**Figure 2.**
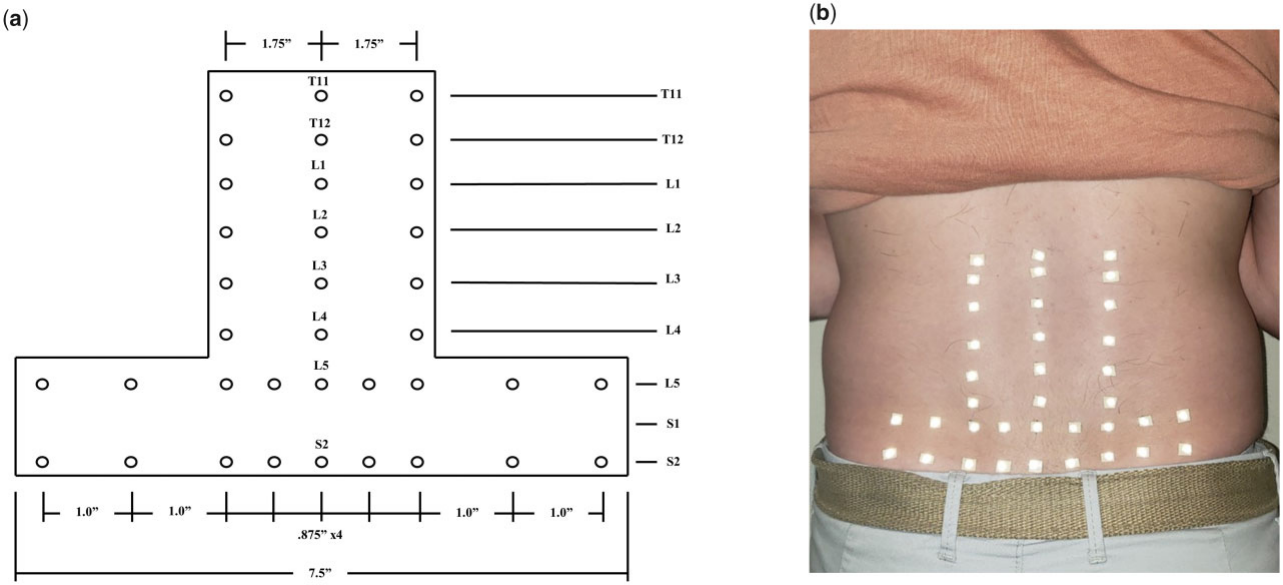
(**a**) Placement of reflective markers for motion-capture study and (**b**) the markers on a subject’s lower back. The skin stretch during different spinal motions was estimated by calculating using the distance between markers.

Participants were asked to perform three repetitions of several single-planar and multi-planar motions. Skin stretch at different areas of the low back was measured during these motions via an array of 36 reflective markers on subjects’ lower backs (see Figure 2) and tracked using a 10-camera motion capture system.

**Figure 3.**
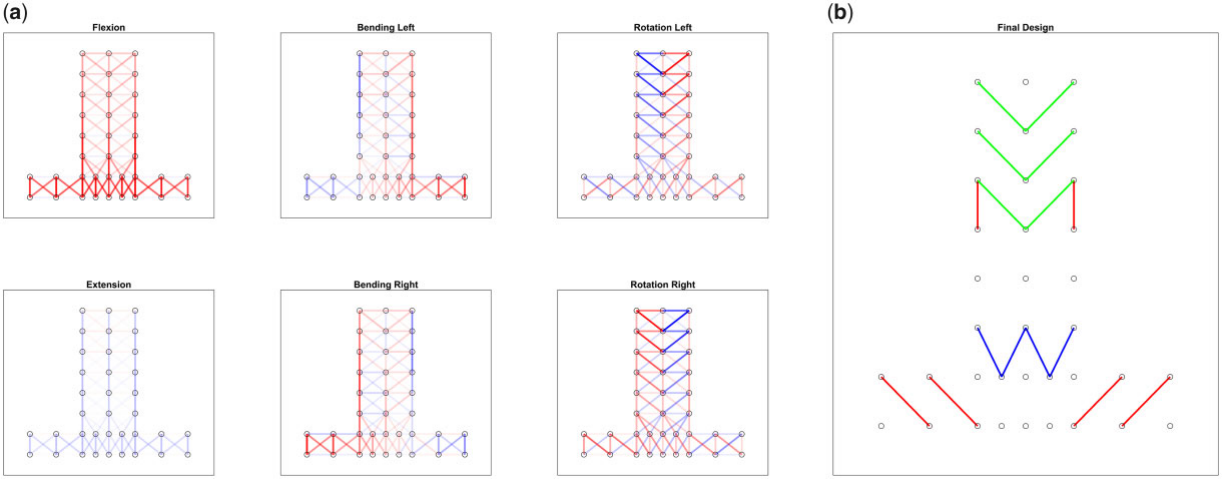
Summary of the extension magnitude for (**a**) each single-planar motion—higher magnitudes of extension indicated by darker shades of red and higher magnitudes of compression indicated by darker shades of blue—and (**b**) the result array design. The reflective markers are indicated by the circles, following the placement depicted in Figure 2.

The engineering strain ($\Delta L/L_{0}$) was calculated between the adjacent reflective markers during each motion. The LoME for each of the six single-planar motions (flexion, extension, lateral bending left, lateral bending right, axial rotation left, axial rotation right) were identified (see Figure 3a). Strain gauge locations were chosen to capture the clearest signal of spinal motion along specific spinal degrees of freedom by considering the LoME and the strain limitations of the gauges (see Figure 3b); four sensors were selected to primarily capture flexion-extension (marked by blue), six sensor locations were chosen to detect lateral bending motion (marked by red), and six sensors were placed to optimally capture axial rotation motion (marked by green).

**Figure 4.**
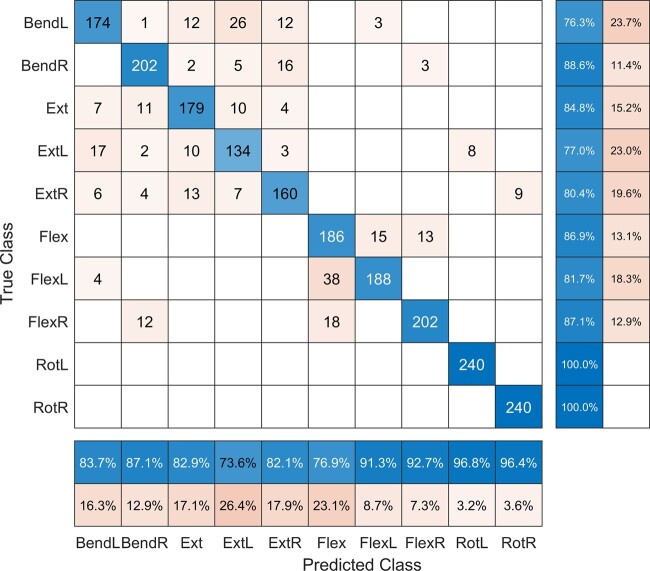
Confusion matrix, depicting the true class of exercise being performed by a subject and the corresponding predicted class, using the skin strains captured by the optimized strain gauge array design as predictors in a machine learning classification model. Predictions along the diagonal represent accurate exercise classifications.

The array design was evaluated by its ability to differentiate between different spinal movements in a support vector classification model. The model input was the maximum skin strain between reflective markers included in the final design (Figure 3b) for each subject-motion combination. The output was the model prediction of which spinal motion was performed. The spinal motions considered in the evaluation included single-planar movements (lateral bending left, lateral bending right, flexion, extension, axial rotation left, and axial rotation right) and multi-planar movements (flexion-left, flexion-right, extension-left, and extension-right). Data from 20 subjects were randomly selected for training the model, and the data from the remaining 8 subjects were selected for evaluating the model. This process of training and testing the model was repeated 10 times. The model achieved an average of 90% classification accuracy (see Figure 4). Ten other designs (randomly generated placements of the 16 strain gauges) were also evaluated using the same methods for comparison, which achieved an average of 76% classification accuracy. None of the random designs achieved greater than 83% accuracy. From this evaluation, we validated that skin stretch can be a reliable metric for evaluating different spinal motions, and that the quality of data for inferring spinal kinematics may be optimized by placing markers along the LoME of the lumbar spine.

During desired applications, the strain gauges must be applied to the lumbar spine to capture skin strains and estimate spinal kinematics. One option is an adhesive layer to hold the strain gauges to the lower back (such as kinesiology tape, which is commonly used in athletics and biomechanical applications). A validation test was conducted to compare the skin strain under normal conditions and when kinesiology tape is applied to the lumbar spine. It was determined using a finite-element model that although an adhesive layer diminishes the strain magnitude, general strain patterns during motion were not significantly affected.^42^ This finding further validated the use of kinesiology tape to adhere the strain gauges to the spine, and further emphasizes the need to place sensors along the LoME to obtain the clearest signal possible.

Finally, a validation tested was performed to evaluate the device’s ability to estimate spinal kinematics. The array of the 16 sensors was adhered to the lumbar spine of a cadaver specimen. The specimen was supported using a harness and manually manipulated in a series of spinal motions along the sagittal plane. The flexion of the cadaver spine was measured using an electromagnetic tracking system, adhered to the L1–S1 functional spinal units via bone pins. A lasso regression model was developed to predict spinal motion as a function of sensor readings root-mean squared error of less than 10% (for full details, see^24^).

### Array implementation

**Figure 5.**
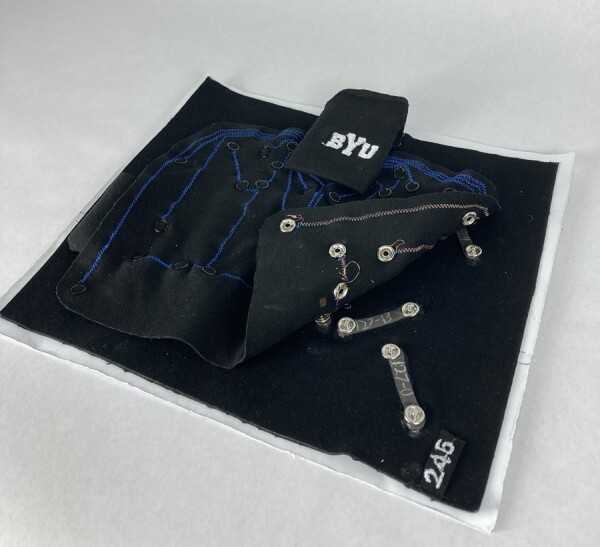
The vertebral motion tracking system of 16 nanocomposite sensors (two are visible from underneath the cover in the lower right side) on kinesiology tape with stitched wiring.

Once the array was designed and its ability to capture spinal motion was shown, its usability in the context of clinical practice was assessed. A prototype of the SPINE Sense System—composed of the 16 nanocomposite sensors adhered to kinesiology tape with a scripted explanation about how the device is used including its integration with its smartphone application (see Figure 5)—was presented to 32 potential users of the device including clinicians and patients with cLBP.^32^

Reviewers were asked to fill out the System Usability Scale, which is a common validated industry standard to evaluate the general usability of a system.^43^ The System Usability Scale score is from 0 to 100—a score above a 68 indicates a device that is intuitive to use.^44^ The mean value of our study usability score was 72, which is considered above average. A more comprehensive breakdown of the results is presented in Table 2. It was concluded from these results that the device is usable and attractive to clinicians and patients who are potential users of the device.

**Table 2.** System Usability Scale scores from clinicians and patients.

Item	Clinicians	Patients	Total
Participants	19	13	32
Mean score	70.79	73.85	72.03
Standard Deviation	13.74	6.69	11.51

The reviewers were also asked open ended question about the design of the device and given the opportunity to voice ideas, questions, and concerns regarding the design of the device. Common feedback and comments about the design of the device included the following:

- The easy application and flat compact design of the device was common positive feedback received.
- The simplicity of the system of its use with a smartphone application was also well received among the potential users.
- Some concerns and questions included the concerns of the device only coming in one size, the duration of adherence, and ease of removal.
- There was also a concern about the tackiness of the kinesiology tape with recommendations of other common preferred athletic tapes.

### Electronics design

**Figure 6.**
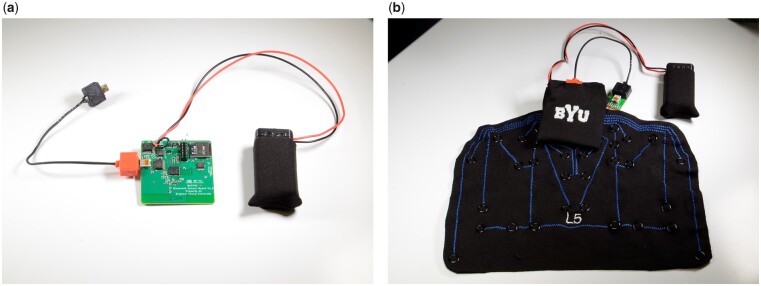
(**a**) Custom PCB for reading the resistance across all 16 piezoresistive sensors and (**b**) integration of PCB with final SPINE Sensor System.

The electronics portion of the system, required to read data from the 16 strain gauges, was designed to be minimal in both size and complexity to facilitate a national clinical study and obtain consistent results across investigation sites. A custom printed circuit board assembly (PCBA) was designed and fabricated with a 16-channel switcher to cycle through all the sensors and sample at a rate of 800 Hz. This PCBA measures 54.61 mm × 50.8 mm (2.15 in × 2 in) and is 4.6 mm (0.181 in) tall at its highest point, with a mass of 12.00 g. The planar, lightweight design enables it to be tucked into a small fabric pouch mounted to the sensor array, without disturbing the subject being tested, adding significant weight, or impacting the motion of the sensor array during exercises. Custom cables using the micro-HDMI connection standard further reduce the size, weight, and complexity of the system. This provides a flexible 17 channel (16 sensors and common ground) connection between the array and the custom PCBA (see Figure 6).

The board is powered by a standard 9 V alkaline battery (nominal mass 45 g), which is readily available across the country. The battery is connected to the custom PCBA using a small power cable and wire (for a total system mass of 64 g), so that the battery can be mounted off to the side of the test subject, while providing multiple hours of charge to the PCBA. A user-friendly switch and LED system allows the clinician to know (*a*) the board is powered on and (*b*) the battery has a good charge and/or needs to be replaced. This simple system reduces the possibility of failure on the part of the electronics and custom PCBA due to battery/charging concerns.

To extract and record sensor readings from the device, a smartphone app was developed for Android^®^ smartphones, and is currently available on the Google Play^®^ app store. The app has an easy-to-use interface, designed to reduce options or confusion for the clinician.

## Subject testing protocol

Using the SPINE Sense System developed as described above, data are acquired from two cohorts (150 healthy, 150 symptomatic) of participants during clinician-guided diagnostic movements, in order to provide a sufficient body of data to identify major spinal movement deviation phenotypes. Subjects are between 35 and 65 years old. This is the age range with the greatest prevalence of cLBP.^45^ Demographic characteristics (sex, age, body mass index [BMI]) are approximately matched across the two cohorts. Data gathering will occur at BYU, along with various partner BACPAC sites, such as University of Pittsburgh, UCSF, OSU, and Harvard. The symptomatic cohort is recruited from individuals who are already scheduled for physical therapy for complaints of cLBP. The exclusion criteria for asymptomatic participants are current or history of lumbar spine pain for which treatment was sought at any point during the participant’s lifetime (“treatment” is defined as having seen a physiotherapist, chiropractor, osteopath, or medical doctor for the condition), known scoliosis and inability to receive MRI (eg, metal or electrical implants, claustrophobia or possible pregnancy). In addition, all participants (symptomatic and asymptomatic) must be able to assume a vertical position and move without an assistive device. Participants are screened for exclusion criteria, and after consent is given, receive our standardized diagnostic movement examination and are scheduled for their MRI.

**Figure 7.**
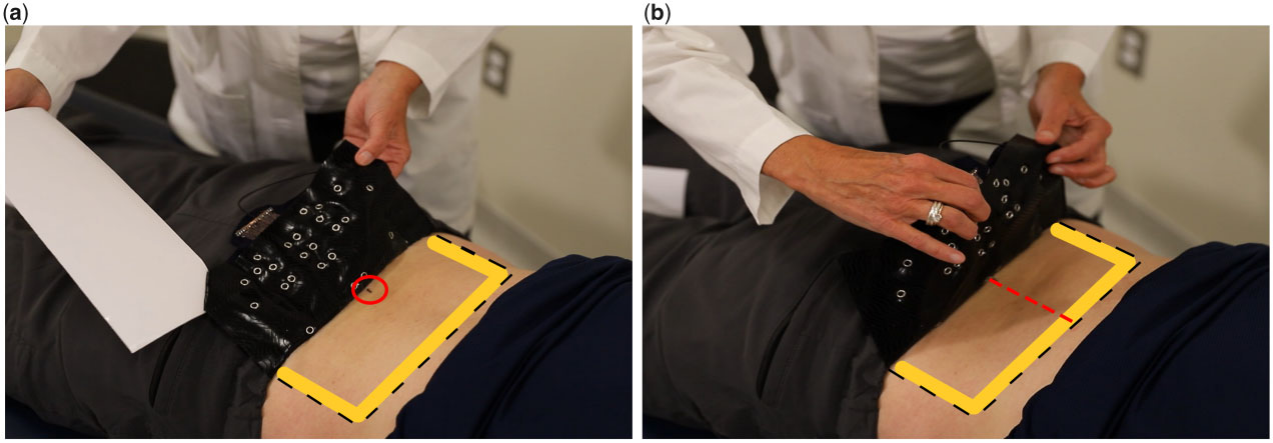
The SPINE Sense System is placed to the lower back using the L5 spinal process unit as a to guide the placement (**a**) and is aligned vertically on the lower back (**b**). The kinesiology tape adherence is reinforced on the edges (depicted in yellow in the figures above) using pre-tape spray.

With the participant in standing position the spinous process of L5 is palpated and marked. The participant then transfers onto a treatment table into prone position. The lower back is sprayed with a commercially available pre-tape spray adhesive and the array is attached as depicted in Figure 7.

**Figure 8.**
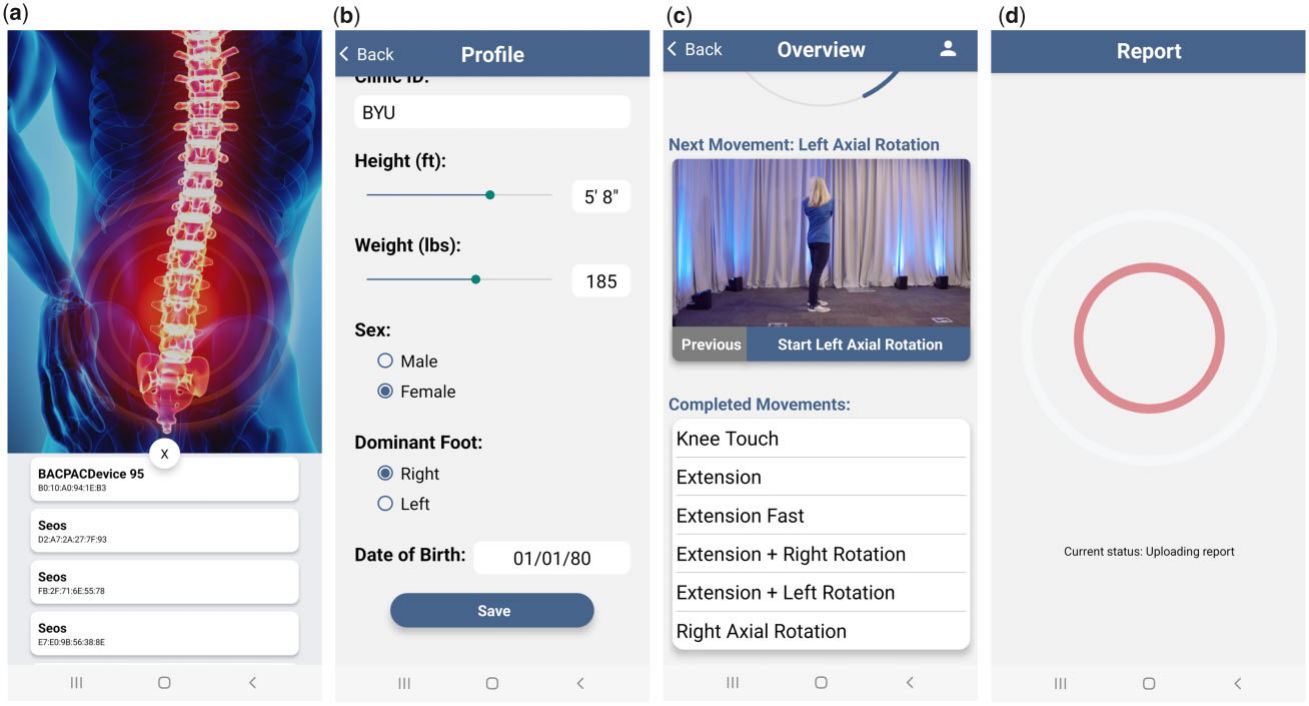
Summary of the SPINE Sense App during the different stages of motion capture. The clinician or participant (**a**) opens the app, (**b**) enters participant demographic information, (**c**) is guided through the series of spinal motions to perform, and (**d**) uploads data to the cloud server.

After the array is adhered, the participant is ready to begin the spinal motion tests. Each movement is performed six times with a 2-second break between repetitions. The clinician demonstrates each movement, and the participant is allowed to practice the movement once. Participants are instructed to perform the motions to the extent possible without inducing pain. The “SPINE Sense” app, available on Play Store, is used to guide the clinician and participant through the spinal motion tests. Once the app is connected to the PCBA via Bluetooth (Figure 8a), it requests basic biometric data along with a de-identified subject ID (Figure 8b). Then, it provides a sequential series of one-click-one-action buttons that first demonstrates the spinal motion for the participant, initiates the recording of data as the subject then performs six repetitions of the movement, and stops recording the data and downloads them via Bluetooth from the PCBA (Figure 8c). Once the data are downloaded, the app continues to the next motion. After all spinal motions are completed and confirmed by the clinician, the app uploads the data to a cloud server via WiFi or cellular internet to be accessed by the researchers for analysis (Figure 8d).

Our project involves classic uniplanar movements (flexion, extension in the sagittal plane, rotation in the transverse plane and side bending in the frontal plane), but also combination of those (flexion and extension with rotation) and a functional sit-to-stand motion. Further details of each movement and the order in which they were performed, are listed below:

1. Knee touch: the participant is instructed to round their lower back and reach with their hands to the respective knees from an upright position.
2. Extension: The participant extends their spine in the sagittal plane as far as comfortably possible.
3. Extension fast: The participant extends their spine similarly, but as fast as comfortably possible.
4. Extension-right: The participant extends their spine in the sagittal plane while rotating it in the transverse plane to their right.
5. Extension-left: The participant extends their spine in the sagittal plane while rotating it in the transverse plane to their left.
6. Rotation-right: The participant crosses their arms over their chest and rotated their spine in the transverse plane toward their right side while maintaining pelvis position (facing anteriorly).
7. Rotation-left: The participant crosses their arms over their chest and rotates their spine in the transverse plane toward their left side while maintaining pelvis position (facing anteriorly).
8. Side bending-right: The participant flexes their spine to their right in the frontal plane while maintaining a forward-facing trunk orientation.
9. Side bending-left: The participant flexes their spine in the frontal plane to their left while maintaining a forward-facing trunk orientation.
10. Flexion-right: The participant flexes their spine and hips in the sagittal plane while rotating the spine in the transverse plane to the right.
11. Flexion-left: The participant flexes their spine and hips in the sagittal plane while rotating the spine in the transverse plane to the left.
12. Up and go: The participant sits on a short stool and rises, without the assistance of their hands and walked two steps forward.
13. Flexion: The participant flexes their spine and hips in the sagittal plane as far as comfortably possible (slight flexion of the knees is allowed).
14. Flexion fast: The participant flexes their spine similarly, but as fast as comfortably possible.

After the motion tests are complete, the SPINE Sense System is removed with the help of paper towels and baby oil. The duration of the test, including the don and doff of the SPINE Sense System, is approximately 15 minutes.^21^

As reported in the Anchor paper, the BACPAC Minimum Dataset is used as patient reported outcomes.^46^ Briefly, the assessment consists of 19 questions/questionnaires that investigate demographics and pain location (radicular/non radicular and if it is widespread), pain intensity, pain duration and frequency, pain interference, pain catastrophizing, pain somatization, physical function, sleep, depression, anxiety, substance use, and opioid use. In addition, our site added a physical activity questionnaire.^47^ Participants are requested to complete the questionnaire during the week prior to the motion-capture study.

## Data analysis

After the PCBA uploads the electrical signal from the 16 piezoresistive strain gauges measured during the set of exercises, the data will be processed, uploaded to the BACPAC Data Portal,^48^ and analyzed. This section will present the methods that will used to analyze said data. Results from early stages of subject testing will also be presented as a preview of anticipated findings.

High deflection strain gauges are a relatively new innovation, and the interpretation of such is a complex task due to the nonlinear output of the sensors.^49^ Past studies have used a variety of curve-fitting analyses and machine learning algorithms to extract biomechanical information from the skin-mounted strain gauges.^36^^,^^50–52^ For the purposes of this research, a model that accounts for the viscoelastic material properties of the sensors was developed to extract stretch magnitude and strain rate (for full details, see^53^) Additional metrics, such as asymmetry of motion, will be extracted by comparing the calibrated sensor outputs on mirror sides of the sagittal plane. These metrics will be calibrated for each participant using the knee touch motion performed during the first stage of subject testing to account for inter-subject variations of subcutaneous tissue and anthropologies. The metrics will then be used to run two general analyses:

1. Biomechanical analysis: Analyze the relationship between spinal kinematics and PROs.
2. Phenotype analysis: Cluster participants by similarity of motion patterns and demographics, identify dominant features of each cluster (ie, the motion-based phenotype), and investigate correlation between phenotype and PROs.

The Biomechanical Analysis will provide a conceptual understanding of how and to what extent spinal kinematics correlate with patient well-being, and the Phenotype Analysis will provide clinicians and patients with information that may facilitate the prescription of more effective treatment paradigms.

### Biomechanical analysis

One objective of this research endeavor is to quantify the extent to which spinal kinematics correlate with different PROs. Past studies have attempted to differentiate between the motion of subjects with chronic and acute low back pain from the motion of asymptomatic subjects, with promising results as described below.^54–57^ This research endeavor will complement previous findings and make further contributions to this topic by analyzing spinal kinematics during a broader range of motions and testing the correlation between spinal kinematics and additional patient-reported metrics. These tests will be accomplished by (1) identifying features of interest that may correlated with PROs and (2) running a series of statistical tests to investigate the correlation.

#### Features of interest

Two obvious biomechanical features relating to cLBP are spinal range of motion for a given exercise and the velocity at which the exercise is performed. Previous studies have found that patients with cLBP have statistically lower ranges of motion and velocities in certain exercises than control subjects.^54^^,^^55^ It is expected that the range will change with pathology. However, there are few other specifically identified biomechanical features that have been correlated with cLBP phenotypes in a quantifiable way. Two recently studied attributes of patient motion include time-of-rise^56^ and jerk level (rate of change of acceleration during a given motion).^57^

In order to provide guidance for the identification of features of the SPINE Sense data, a recent study was undertaken by the BACPAC Technology Site at Brigham Young University using participants with cLBP and control subjects who did not suffer from cLBP, that followed the same set of exercises described above (3. Subject Testing Protocol). The participants were fitted with three inertial measurement units (IMUs) on the sacrum, L4, and C7 spinal segments. Kinematics including acceleration, angular velocity and jerk were recorded and are currently being analyzed for motion features that most clearly identify cLBP (eg, features with high variance across the subject set).

Other potential metrics available from the SPINE Sense system include asymmetry of motion, a range of other derivative metrics from strain, time, and surface shape features of the back (such as angular velocity, back curvature, etc.), as well as changes in metrics with repeated exercises (such as variance and gradients).

#### Statistical tests and regression models

**Figure 9.**
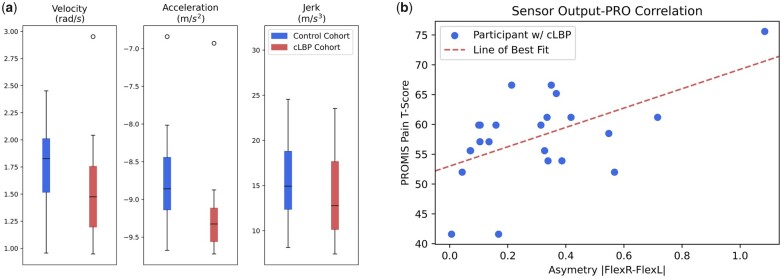
(**a**) An example of a comparison between the velocity, acceleration, and jerk between 16 control subjects (blue) and 28 subjects with cLBP (red) during Flexion Left collected from the IMU placed on the C7 (*P* values of 0.26, 0.08, and 0.60 for velocity, acceleration, and jerk respectively) and (**b**) scatterplot of asymmetry of motion during flexion -estimated by the absolute value of the difference in the sensor readings during the Flexion Left and Flexion Right motions—and the PROMIS Pain T-score for participants with cLBP (blue dots) with the corresponding best fit line (red).

Statistical tests will evaluate the difference between the spinal motion subjects with cLBP and asymptomatic subjects, as well as the motion of subjects who do and do not take opioids. Student *t* tests are ideal for this analysis because cLBP status and opioid use are binary, categorical variables (ie, a subject either experiences cLBP or does not). An example of the results obtained from Student *t*-test analyses conducted on preliminary data collected with the IMU placed on the C7 of the spine during flexion left is depicted in Figure 9a. The Bonferroni correction will be applied in the statistical analyses to account for the multiple comparisons that will be conducted. Quantitative patient-reported outcomes (ODI, PROMIS, and IPAQ scores) will be correlated with spinal kinematics by running several regression analyses. Multiple linear regression models will be developed to predict PROs as functions of spinal kinematics and demographics. Model features will be selected using Stepwise Regression techniques (a hybrid of forward and backward feature selection). The number of features included in the final models will be determined by evaluating the adjusted R-squared of each model with different number of explanatory variables. A sample of the correlation between a single kinematic measurement and a PRO is depicted in Figure 9b.

The IPAQ questionnaire categorizes persons’ activity levels into one of three bins: low, medium, and high. This data format is well suited for F-statistic and ANOVA tests to investigate whether physical activity is correlated with spinal kinematics.

It is anticipated that the results of these analyses will provide clinicians and researchers with an understanding of how spinal kinematics can be used to interpret the different metrics of patient well-being, to what extend spinal kinematics correlate with PROs, and which features are most informative in assessing a subject’s condition.

### Phenotype identification and interpretation

The primary objective of this research endeavor is to identify and analyze movement phenotypes of spinal motion. Different etiologies of acute cases of low back pain are often associated with specific changes in spinal motion and specific treatment paradigms exist.^58–66^ It is therefore reasonable to suppose that patients with cLBP who exhibit similar abnormalities in spinal motion respond similarly to a certain treatment.^17^ Objectively identifying and analyzing these motion patterns may lead to more personalized treatment paradigms and improved outcomes for patients suffering from cLBP.^67^ Phenotype identification will be accomplished via an unsupervised clustering analysis. The approach of this endeavor involves three steps:

1. Cluster participants (from both the symptomatic and asymptomatic cohort) according to spinal motion characteristics observed during the standard exercise routine and demographics by their respective phenotypes.
2. Rank the features of each cluster to determine the features most useful for phenotype segmentation, identify the predominant characteristic(s) of each cluster/phenotype, and quantity the difference of dominant features between clusters.
3. Analyze the relationship between phenotype cluster and patient-report outcomes using one-way analysis of variance (ANOVA) statistical tests.

#### Phenotype identification

The clustering analysis entails selecting which features ought to be included in determining phenotypes, choosing an appropriate algorithm for grouping participants with similar features together, and determining the optimal number of clusters that will be conveyed to clinicians and patients.

##### Features of interest

Several features will be included in the clustering analysis to identify phenotypes among subjects with cLBP; they are:

- Spinal kinematics collected from the skin-mounted sensors. The kinematic measurements of spinal motion that most clearly differentiate between the motion of cLBP and asymptomatic subjects will be prioritized in the clustering analysis (see 4.1 Biomechanical Analysis).
- Subject demographics as collected from the questionnaires.
- Anatomical assessment of the lumbar spine as determined by a radiologist from MRI scans. Although MRI scans of the spine are insufficient on their own to provide adequate detail regarding a patient’s condition to indicate the optimal treatment paradigm,^7^ evidence suggests that MRI images still provide valuable information (such as ailment severity,^68^ which has the potential for clinical application^69^) when considered in conjunction with additional metrics. The presence of specific anatomical anomalies will be included as binary variables (eg, degenerated intervertebral disc in the lumbar spine—0 if no, 1 if yes).

Each feature will be normalized to objectively compare its impact on the final phenotype identification. A principal component analysis will then be used to reduce the number of data dimensions and eliminate feature collinearity.

##### Clustering algorithm

The objective of the phenotype identification is to group together datapoints (ie, participants) that exhibit similar features. Clustering analyses are ideal for identifying predominant spinal-motion phenotypes because the algorithms will dissect data such that the variance between clusters is maximized, while the variance within a cluster is minimized (ie, the clusters are dissected along the natural segmentation in the data).^70^ Hierarchical clustering is well suited for the phenotype-identification analysis due to its ability to cluster both quantitative features (such as spinal kinematics) and categorical features (such as demographics and MRI features) in the same data set.

**Figure 10.**
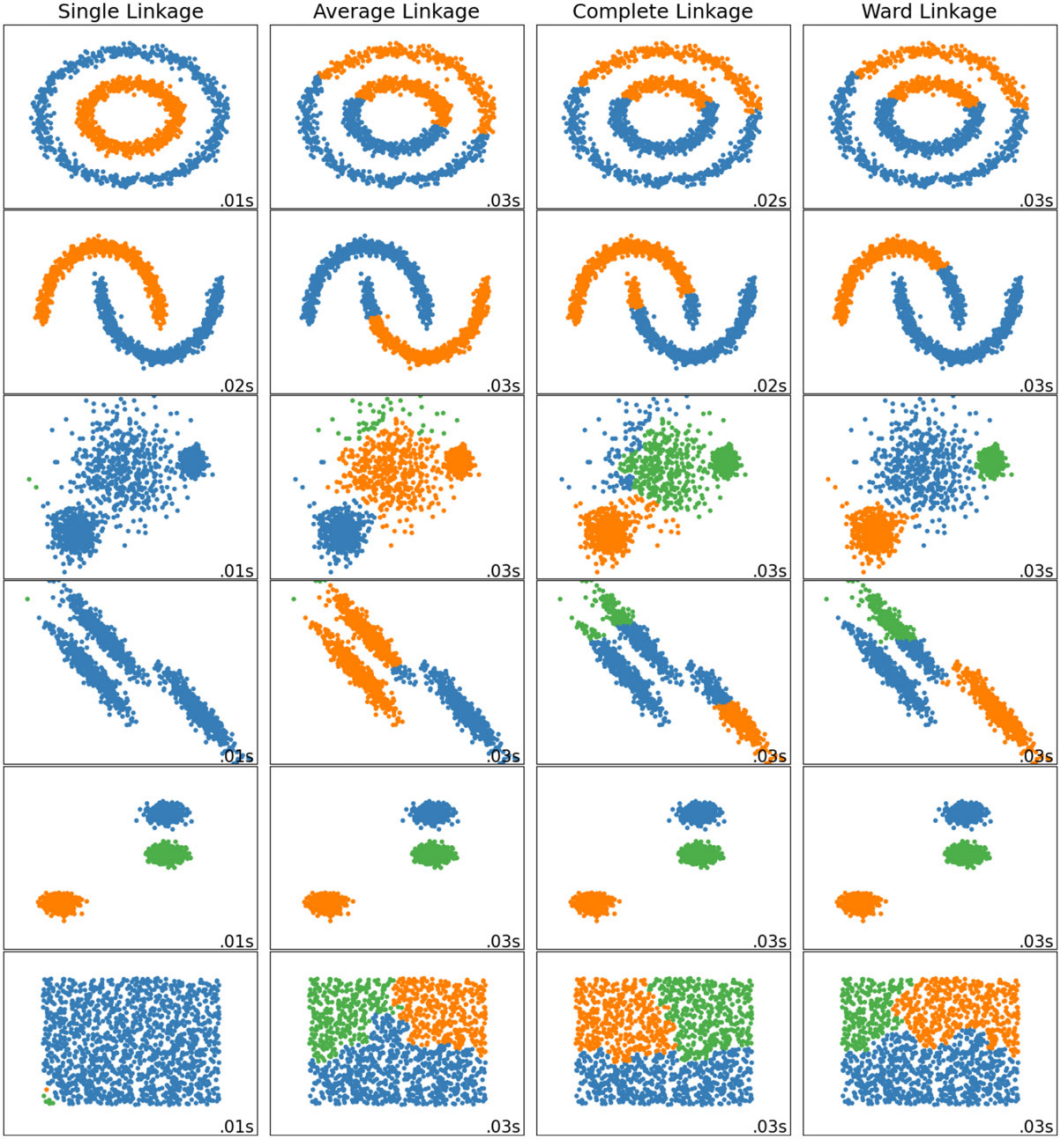
A depiction of how different linkage methods perform for hypothetical data sets, obtained from.^71^ Single Linkage performs well on non-globular data, but performance is diminished by noisy data. Average Linkage and Complete Linkage perform well on data that exhibits clean separations between clusters, but the quality of the results is diminished if the separations are not as distinct. Ward Linkage is optimal when clustering noisy data and will be implemented in this endeavor.^72^

Hierarchical clustering will yield different results depending on the linkage type, which is used to quantify similarity/dissimilarity between data points (Figure 10, obtained from,^71^ depicts how different linkage types employed in clustering analyses cause different results for several hypothetical data set). Due to its robust nature, the Ward Linkage will be implemented in the phenotype analysis.^72^

##### Determining the desired number of clusters

**Figure 11.**
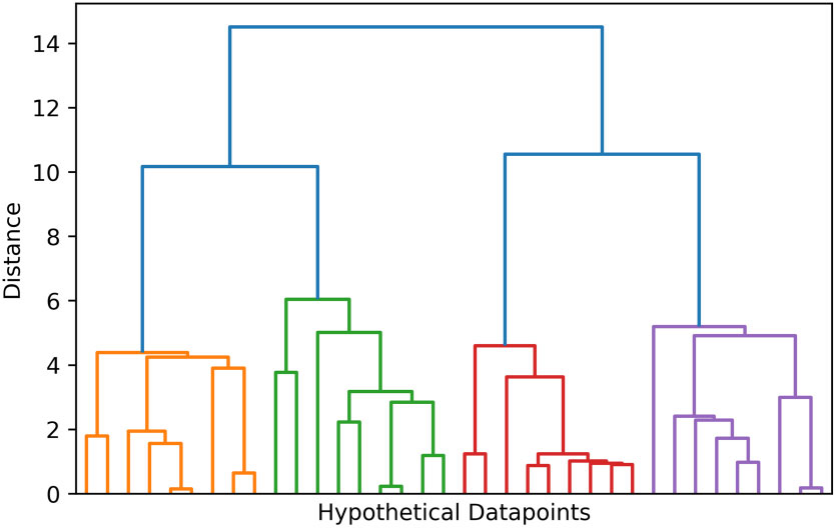
An example of a cluster dendrogram (patterned after original from^73^) By using a cutoff distance score of 8, three clusters were extracted from the data, marked by orange, green, red, and purple. A cutoff distance score of >8 would have resulted in fewer clusters with more observations per cluster, and a cutoff distance score of <8 would have resulted in more clusters with fewer observations.

One decision during the clustering process involves determining the desired number of clusters. An example of how the results of a clustering algorithm may vary according to the number of clusters into which a dataset is segmented is depicted by the dendrogram in Figure 11 (generated from a hypothetical data set). The height of the dendrogram tree demonstrates the dissimilarity score between pairs of observations. A higher dendrogram cutoff score will result in fewer clusters with more observations per cluster. Lower dendrogram cutoff scores will result in more clusters with fewer observations per cluster.

**Figure 12.**
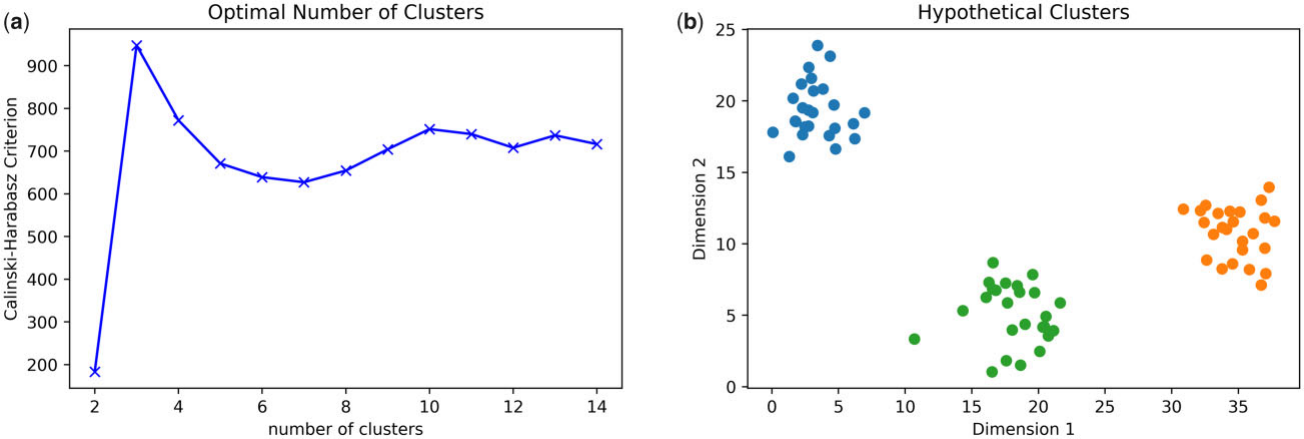
(**a**) The Hypothetical data set is composed of data normally distributed around three centers and (**b**) the corresponding Calinski-Harabasz Criterion for 2 through 14 clusters. The Calinski-Harabasz score is the highest when the number of clusters is three, indicating three clusters provide the clearest segmentation between groups of datapoints.

The objective to minimize the variance within clusters and maximize the variance between clusters can be quantified using the Calinski-Harabasz criterion.^70^ Higher Calinski-Harabasz scores indicate better segmentation of the data. The Calinski-Harabasz score will be calculated for a wide range of potential targeted number of clusters, and the number of clusters that result with the highest score will be implemented in the final analysis. This will provide clinicians with the clearest distinction between participant phenotypes. A hypothetical dataset and the corresponding Calinski-Harabasz scores at a variable number of clusters is depicted in Figure 12.

#### Feature ranking—phenotype description

While a principal component analysis of the original dataset will be used to remove the collinearity between features and reduce the number of dimensions in the clustering analysis, the resulting phenotypes will be described in terms of the original biomechanical features to preserve interpretability for clinicians and patients. The normalized original feature values will be averaged for each cluster. The difference between average feature values will be calculated for each pair of clusters, which will indicate which features had the largest effect in differentiating the phenotypes. This will enable us to rank feature importance for each cluster and interpret the dominate subject characteristics (eg, subjects from cluster A demonstrated limited motion during lateral bending exercises, while subjects from cluster B experience high levels of anxiety, etc.).

Additionally, this feature ranking will enable us to determine which features had the least impact on distinguishing between clusters. We can make future analyses more efficient by neglecting the features of little consequence in future studies.

#### Clinical significance of phenotypes

**Figure 13.**
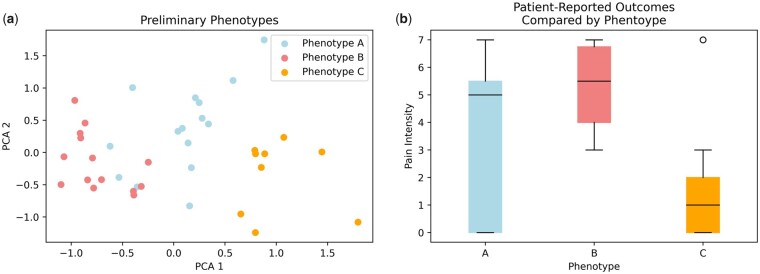
An example of the results of a phenotype identification analysis and its corresponding clinical interpretation from preliminary data. (**a**) Depicts the different phenotypes by color on the first two principal components of the motion and demographic data. Phenotype A was comprised of participants who exhibited significantly different velocities between their flexion right and flexion left motions (ie, performing flexion towards one side was much faster than the other); Phenotype B was comprised of mostly participants with cLBP, high BMI scores, and who performed flexion right and flexion left with similar velocities; and Phenotype C was comprised of mostly asymptomatic patients with low BMI scores and a dominant side during lateral bending. (**b**) Depicts boxplots of the Pain Intensity scores for the participants, grouped by phenotype. A one-way ANOVA test calculated an F-statistic of 7.23 and a corresponding *P* values of 0.002 comparing the Intensity scores between the phenotypes.

Once the phenotypes are distinguished, and the dominant features of each phenotype are determined, we will analyze the relationship between phenotypes and the PROs. It is our hypothesis that some phenotypes will be associated with more severe symptoms and lower quality of life, while other phenotypes will be associated with positive PROs scores and a higher quality of life. This hypothesis will be tested using one-way ANOVA tests to evaluate whether the PROs associated with the different phenotypes significantly differ one from another. As multiple PROs will be tested, the Bonferroni correction factor will be implemented to mitigate the risk of Type I error. An F-statistic with a *P* values of <.05 (after implementing the Bonferroni correction factor to account for multiple comparisons) will be considered statistically significant. An example of a preliminary phenotype analysis (conducted on data from the first 40 participants—27 symptomatic and 13 control) and its corresponding implicated regarding Pain Intensity scores (on a scale of 0–10) from the participants in each phenotype is depicted in Figure 13.

It is also our expectation that each phenotype will be dominantly composed of either control or symptomatic participants (ie, there will be little mixing of controls in any given phenotype). Exceptions to this supposition may indicate a participant is on the verge of transitioning between chronic low back pain and asymptomatic status (ie, participants who experience cLBP but exhibit phenotypes typical of asymptomatic participants may indicate that they have managed their chronic pain well and thereby mitigated its severity).The PRO scores of participants with cLBP who exhibit “asymptomatic phenotypes” will be compared with the PRO scores of all other participants with cLBP using Student t-tests, applying a cutoff *P* values of .05 before implementing the Bonferroni correction factor. Likewise, an asymptomatic participant who exhibits a phenotype typical of participants with cLBP may indicate that the asymptomatic individual is performing suboptimal spinal motions and could benefit from preventative treatment. The PRO scores from these groups will also be compared using Student t-tests.

## Discussion

Although many people suffer from cLBP, treatment efficacy is limited in part due to the difficulty of pinpointing the etiology of chronic and other types of non-specific LBP using the conventional methods of taking static images of the spine. A potential alternative method of assessing spinal health through an analysis of spinal motion and movement patterns. Previous studies have investigated the effects of cLBP on spinal motion and found significant differences between the motion of subjects who do and do not experience cLBP symptoms.^54–57^ Other studies have attempted to leverage this phenomenon for clinical application by tailoring treatments according to patient spinal motion patterns with promising results, but there are currently still insufficient data that warrants validation and widespread clinical implementation.^17^ However, more quantitative analyses of spinal motion could lead to more objective classification methods that are robust to clinical subjectivity^74^ and provide clinicians with the information needed to prescribe more personalized treatments and facilitated improved treatment outcomes.^21^

In order to acquire the requisite objective and quantitative biomechanical insights relating to cLBP, this protocol manuscript presents (1) the development and validation of a spinal-motion monitoring device that is economical and practical for clinical and personal implementation—the SPINE Sense System—and (2) the methods that will be used to analyze the spinal kinematic data to provide clinicians and patients with valuable information in assessing and treating cLBP.

Spinal kinematics will be estimated via an array of high-deflection, inversely piezroresistive strain gauges. These sensors were optimally placed on LoME of the lower back to extract the clearly and most reliable signal from the sensors during different motions. This design was validated by assessing the ability to interpret exercise type using skin-strain predictors with 90% accuracy. A model to predict spinal motions as a function of sensor output was developed using spinal motion data from an electromagnetic tracking system, adhered to a cadaver spine via bone pins. The model provided an estimate of the spinal kinematics along the sagittal plane with a root-mean squared error of less than 10%.^24^ This device—the SPINE Sense System—has already been presented to prospective users among clinicians and cLBP patients, who rated the appliance as user-friendly.^32^

As a result of the provided feedback from the study, future iterations of the SPINE Sense System will be produced in multiple sizes to produce better fit for users. A corresponding phone application has been developed to provide an intuitive and efficient means of storing and transferring data collected from the sensor array.

Data collected from the SPINE Sense system device will be used to validate previous research findings and provide additional information by analyze data from a broader range of single- and multi-planar spinal functional movements. Regression models will be developed to quantify the correlation between spinal kinematics and additional PROs, including opioid-use, anxiety, self-assessed disability, and activity level. Finally, an unsupervised machine learning clustering algorithm will be implemented to identify distinct spinal-motion phenotypes. Additional statistical tests will analyze the relationship between subject phenotype and PROs to provide additional information regarding the clinical significance of each phenotype cluster. the anticipated outcomes of this study will include (1) a set of identified movement-based and demographical features that are most important in the clustering of phenotypes, (2) the phenotype identification of each participant in the study, and (3) initial observations of the relationship between biomechanical response and PROs that may guide treatment response for patients with cLBP going forward. Future validation regarding the clinical significance of the diagnosed phenotypes may include treatment-based classification studies to evaluate which treatment paradigms are best suited for patients in each phenotype.

It is our hypothesis that this information regarding subject phenotypes and their corresponding descriptions will enable clinicians to select treatment paradigms individually tailored according to patient conditions. This in turn will improve treatment efficacy and the quality of life for the millions of patients suffering from cLBP worldwide.
